# A comparison of the mitochondrial proteome and lipidome in the mouse and long-lived Pipistrelle bats

**DOI:** 10.18632/aging.101861

**Published:** 2019-03-19

**Authors:** Amelia K. Pollard, Thomas L. Ingram, Catharine A. Ortori, Freya Shephard, Margaret Brown, Susan Liddell, David A. Barrett, Lisa Chakrabarti

**Affiliations:** 1 School of Veterinary Medicine and Science, University of Nottingham, Sutton Bonington LE12 5RD, UK; 2 School of Biosciences, University of Nottingham, Sutton Bonington LE12 5RD, UK; 3 Centre for Analytical Bioscience, School of Pharmacy, University of Nottingham NG7 2RD, UK; 4 West Yorkshire Bat Hospital, Otley, West Yorkshire LS21 1AJ, UK; 5 MRC-ARUK Centre for Musculoskeletal Ageing Research, UK

**Keywords:** mitochondria, bats, proteomics, lipidomics, FABP3, free fatty acid

## Abstract

It is accepted that smaller mammals with higher metabolic rates have shorter lifespans. The very few species that do not follow these rules can give insights into interesting differences. The recorded maximum lifespans of bats are exceptional - over 40 years, compared with the laboratory mouse of 4 years. We investigated the differences in the biochemical composition of mitochondria between bat and mouse species. We used proteomics and ultra-high-performance liquid chromatography coupled with high resolution mass spectrometry lipidomics, to interrogate mitochondrial fractions prepared from *Mus musculus* and *Pipistrellus pipistrellus* brain and skeletal muscle. Fatty acid binding protein 3 was found at different levels in mouse and bat muscle mitochondria and its orthologues were investigated in *Caenorhabditis elegans* knock-downs for LBP 4, 5 and 6. In the bat, high levels of free fatty acids and N-acylethanolamine lipid species together with a significantly greater abundance of fatty acid binding protein 3 in muscle (1.8-fold, *p*=0.037) were found. Manipulation of fatty acid binding protein orthologues in *C. elegans* suggest these proteins and their role in lipid regulation are important for mitochondrial function.

## Introduction

Recent figures indicate that 21% of the world’s population will be aged 60 or over by 2050 [1]. Determining the mechanisms underpinning the ageing process is vital so that we can improve healthspan to match these increased years of life. In humans, the brain and skeletal muscle both deteriorate as part of the ageing process and mitochondrial dysfunction has been implicated to play a critical role in the age-related decline of these tissues [2–4].

Some common observations have been used to generate theories of ageing. *The rate of living theory of ageing* proposes to explain the variation in mammalian lifespans, it states that lifespan and metabolic rate are inversely correlated [5]. Another theory is the *mitochondrial free radical theory of ageing*, this states that animals with high basal metabolic rates will generate greater levels of reactive oxygen species (ROS) and due to their detrimental effect, have shorter lifespans [6]. Observed mammalian biology mostly aligns with these theories however there are a few notable exceptions, including the naked mole rat and microbats, that live much longer than their small body size and high metabolic rates would predict [7]. The naked mole rat has given insight into mechanisms that prevent cancer, considered to be a disease of ageing [8] [9]. Microbats are exceptionally long-lived considering their small body size and high metabolic rates [10]. For instance, the maximum lifespan for the bat; *Myotis lucifugus* (weight ~8g) is 34 years. In comparison the maximum lifespan of a mouse (*Mus musculus*) (weight ~30g) is 4 years [11]. Bats have been shown to expend double the amount of energy in comparison to non-flying eutherian mammals and yet they live on average three times longer than non-flying eutherian mammals [12]. This raises the question; how do bats maintain such high metabolic rates without succumbing to accumulating damage over their lifespan?

Oxidative stress has been a key focus for the majority of studies investigating longevity in bats. Mitochondrial dysfunction is evident in ageing and age-related diseases. Mitochondrial DNA (mtDNA) is considered to be more susceptible to mutagenesis due to the close proximity of mtDNA to ROS and also the high number of direct repeat regions (8-10 bp) which are prone to deletions [13]. Bat mtDNA was found to have a lower number of repeat regions compared with other mammals [14]. A study by Brunet Rossini compared the production of free radicals in bats (*Myotis lucifugus),* shrews (*Blarina brevicauda*) and white-footed mice (*Peromyscus leucopus*). Bats were found to produce half to one third of the amount of hydrogen peroxide per oxygen molecular consumed compared to both shrews and mice [15]. Research thus far indicates that bats produce less ROS and may also be more resistant to oxidative stress [15].

Hibernation has also been suggested to contribute to longevity in bats, however, when comparing the life span of non-hibernating bats to non-flying eutherian mammals, bats still live on average 7 years longer [11]. Interestingly, during hibernation the fibre composition of the pectoralis muscle of the *M.lucifugus* remains unchanged [16]. Deterioration of cognition coupled with the functional loss of skeletal muscle strength are common features of ageing [17–19]. Studying the mechanisms by which dormant animals, such as bats, prevent muscle atrophy may develop our understanding of age associated muscle loss.

An active mitochondrial population is considered to be an intrinsic cellular requirement for healthy ageing. Loss of mitochondrial functionality has been implicated in neurodegenerative diseases as well as in age related sarcopenia [2,20]. Previously, we have interrogated skeletal muscle and brain tissue mitochondria profiles in young and middle-aged mice and shown that there are characteristic, measurable differences in proteins and lipids [21,22]. There are few published studies investigating ageing in bats, and research on captive populations in the UK is limited by their protected status [10,23,24]. In this study we compared the mitochondrial lipidome and proteome of whole brain and skeletal tissues from adult *Pipistrelle* bats (maximal lifespan 12 years) with parallel sample types prepared from young and middle-aged *Mus musculus* [21,22].

## RESULTS

### The bat and mouse brain mitochondrial proteomes are distinctly different

**Figure 1:**
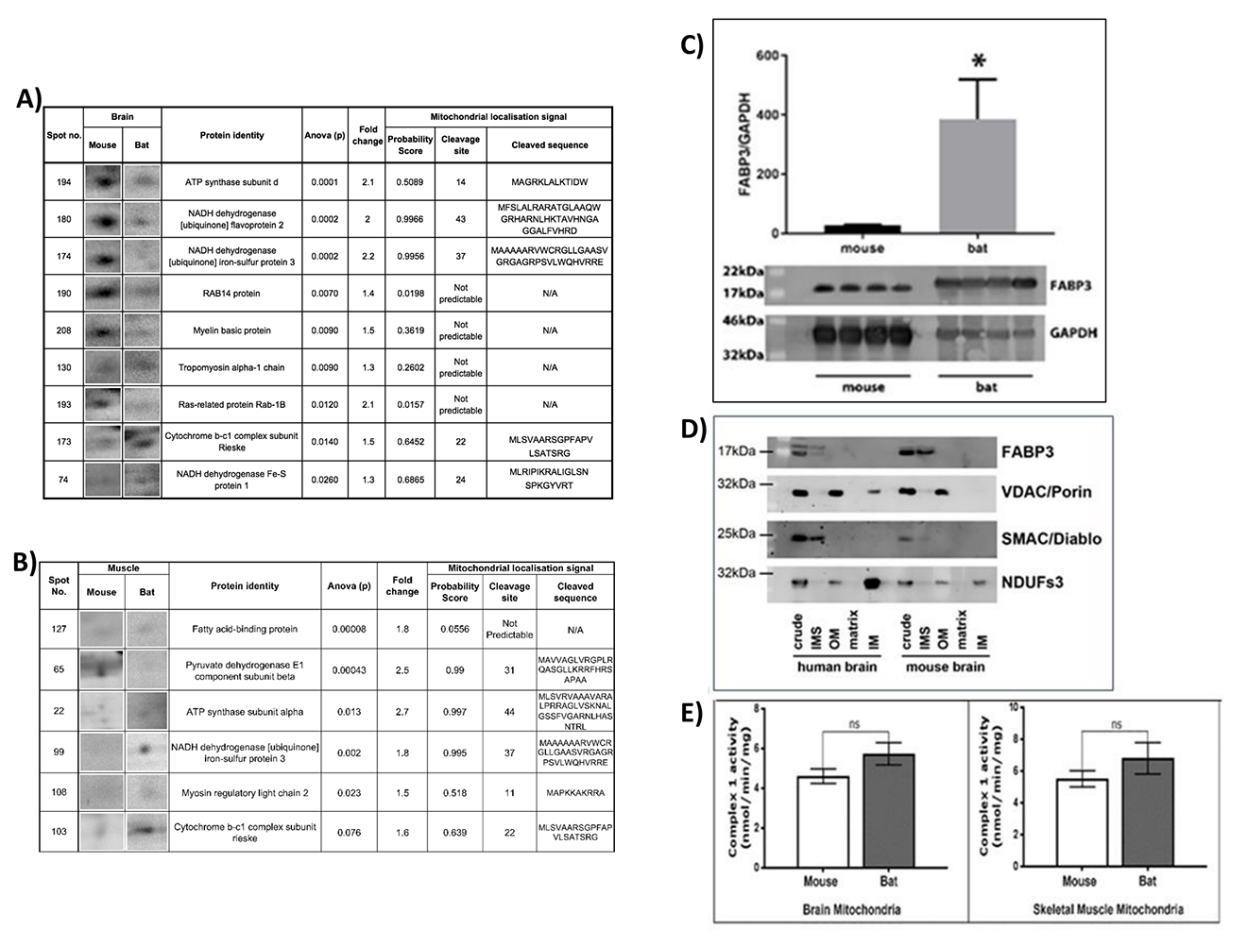
**Proteomics analysis of mouse and bat mitochondrial fractions from brain and muscle tissues.** Identification of protein changes between the mouse and bat brain mitochondrial proteome. (**A**) Ten protein spots were selected after (SameSpots) analysis comparing mouse brain mitochondria aged (4-78 weeks (n=8) and bat brain mitochondria (adult, n=4). The table shows the identities of the proteins (MASCOT) and statistical analyses (one-way ANOVA) for each protein spot. The mitochondrial localisation probability, predicted cleavage site and sequence were calculated for each protein using Mitoprot. Identification of protein changes between the mouse and bat skeletal muscle mitochondrial proteome. (**B**) Six protein spots were selected after (SameSpots) analysis comparing mouse brain mitochondria aged (4-78 weeks (n=6) and bat brain mitochondria (adult, n=6). The table shows the identities of the proteins (MASCOT) and statistical analyses (one-way ANOVA) for each protein spot. The mitochondrial localisation probability, predicted cleavage site and sequence were calculated for each protein using Mitoprot. (**C**) Fatty Acid Binding Protein 3 (FABP3) levels are significantly higher in the adult bat muscle mitochondria when compared with adult mouse muscle mitochondria. Skeletal muscles were prepared to provide enriched mitochondrial fractions from four adult (>1 year) bats and four adult (12 weeks) mice. Western blotting with an FABP3 antibody confirmed the difference in this protein in mitochondrial samples from the different species. GAPDH antibody was used on the same blot to provide a control for band density normalisation. (**D**) FABP3 is localised to the mitochondrial inter-membrane space. Antibody to FABP3 was used to detect its presence in sub-mitochondrial preparations from mouse and human brain mitochondria fractions. In both species FABP3 was detected in the inter-membrane space and correlated with the presence of SMAC/Diablo, a specific protein marker for this compartment. (**E**) Complex 1 activity in the bat and mouse mitochondria. Complex 1 was measured spectrophotometrically in the bat and mouse brain mitochondria. The activity of complex 1 is higher in the bat brain mitochondria however this did not reach significance (*p*=0.094). Six biological replicates for the mouse brain mitochondria and four biological replicates for the bat brain mitochondria, two measurements were taken per sample. Complex 1 was also measured spectrophotometrically in the bat and mouse skeletal muscle mitochondria. The activity of complex 1 is higher in the bat skeletal muscle mitochondria however this was not significantly different. Eight biological replicates for the mouse brain mitochondria and four biological replicates for the bat brain mitochondria, two measurements were taken per sample. All assays contained 30mg/ml mitochondrial protein. Columns display mean activity ± SEM. A two-tailed *t-*test with Welch’s correction was performed, ns=no significant difference.

We wished to gauge whether the biochemical composition of mitochondria in bat brain is markedly different to mitochondria in the mouse brain. We report the top nine proteins that have significantly different relative quantities when comparing the bat and mouse brain mitochondrial proteomic profiles (Figure 1A). Quantities of tropomyosin alpha-1 chain, cytochrome b-c1 complex subunit Rieske and NADH dehydrogenase iron-sulphur protein 1 are relatively higher in the bat brain mitochondrial proteome compared to the mouse (*p*<0.05). Whilst, ATP synthase subunit d, NADH dehydrogenase flavoprotein 2, NADH dehydrogenase iron-sulphur protein 3, RAB14, myelin basic protein and Ras-related protein are each significantly relatively more abundant in the mouse brain mitochondrial proteome (*p*<0.05). We measured more ATP synthase subunit d (2 fold) in the mouse brain mitochondrial proteome in comparison to the bat (*p*=0.0001). Mitoprot probability scores for mitochondrial import are calculated for spot numbers 194, 180, 174, 173 and 74. The proteins identified from these spots have sequence motifs that support a localisation in the mitochondrial compartment.

### The bat and mouse skeletal muscle mitochondrial proteomes are distinctly different

We have highlighted the top six proteins that have significantly different relative quantities when comparing the bat and mouse skeletal muscle mitochondrial proteomes (Figure 1B and C). ATP synthase subunit alpha, cytochrome b-c1 complex subunit Rieske, myosin regulatory light chain and NADH dehydrogenase iron-sulphur protein 3 are each relatively more abundant in the bat skeletal muscle compared to the mouse skeletal muscle mitochondria. Each of these proteins is also predicted to localise in the mitochondrion and have characteristic cleavage site sequences for mitochondrial import. Interestingly the proteins cytochrome b-c1 subunit Rieske and NADH dehydrogenase iron-sulphur protein 3 were also identified in the brain mitochondria comparison. There is significantly more cytochrome b-c1 complex subunit Rieske in the bat skeletal muscle (1.6-fold), comparatively more of this protein was also found in the bat brain mitochondria. In both the mouse brain and skeletal muscle levels of NADH dehydrogenase iron-sulfur protein 3 were found to be over two-fold different when compared with the bat, in skeletal muscle mitochondria there is more protein in bat samples whereas in the brain the NADH dehydrogenase iron-sulfur protein 3 is found in greater quantities in mouse mitochondria (1A).

Pyruvate dehydrogenase E1 component subunit beta is significantly more abundant in the mouse skeletal muscle mitochondria by a fold change of 2.5. Pyruvate dehydrogenase E1 component subunit beta is a mitochondrial protein and the cleaved sequence is included (Figure 1B). Fatty acid binding protein 3 is more abundant in bat skeletal muscle with a fold change of 1.8. However, a predicted cleavage site in FABP3 was not identified using Mitoprot. FABP3 is a small protein that may passively cross the outer mitochondrial membrane, we confirmed and refined its mitochondrial localisation by western blotting sub-mitochondrial fractions derived from human and mouse brain enriched mitochondrial samples (Figure 1D). Analysis of mitochondrial sub-fractions localised FABP3 to the intermembrane space in both human and mouse.

### Fatty acid binding protein 3 relative quantities are significantly different from the mouse in the bat mitochondria

We confirmed our proteomics data, measuring significantly higher levels of FABP3 in the bat skeletal muscle mitochondria by western blot (*p =* 0.037) (Figure 1B and C).

### Comparative differences in the mitochondrial proteome are not reflected in mitochondrial complex 1 activity

Many of the differences between mitochondria when comparing bat with mouse were in proteins associated with the electron transport chain. We wanted to know whether these differences grossly change the function of complex 1. We measured complex 1 activity in muscle and brain mitochondria from each species. Though in each tissue the complex 1 activity was found to be lower in the mouse the differences were not significant (Figure 1E).

### Mitochondrial lipid composition is characteristic for species and tissue type

**Figure 2:**
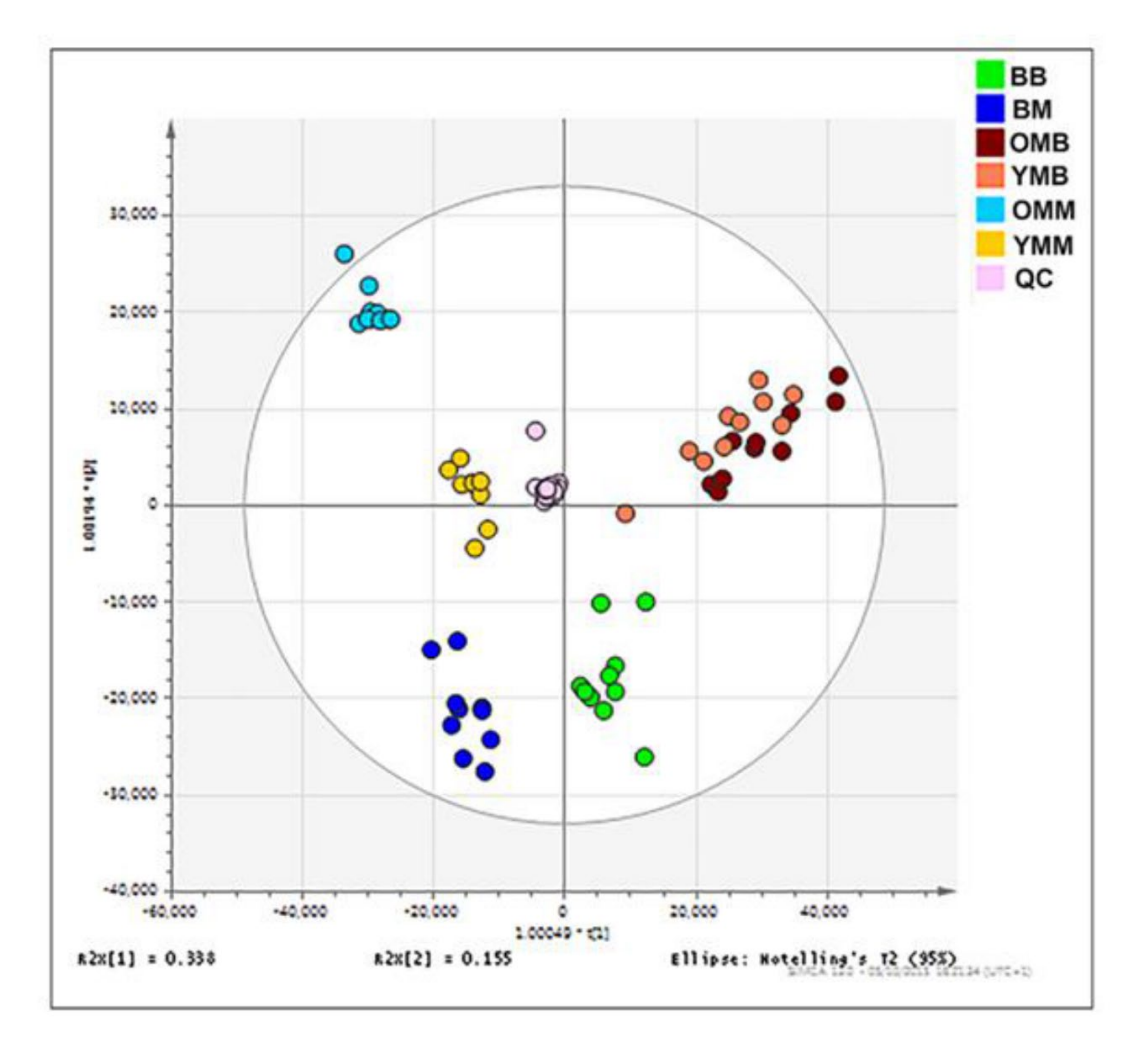
**Mitochondrial lipid composition differs between the bat and mouse mitochondrial proteomes**. Orthogonal partial least square-discriminant analysis (OPLS-DA) of lipids found in the brain and skeletal muscle mitochondria from the mouse and the bat. Separation across the x-axis is according to tissue type with the skeletal muscle mitochondrial samples congregating to the left quadrants and the brain mitochondrial samples to the right. Along the y-axis separation delineates mammalian species with the bat mitochondrial samples grouping at the lower quadrants and the mouse mitochondrial samples grouping at the upper quadrants. Bat brain (BB) mitochondrial samples (adult, n=10) are shown on the OPLS-DA by the green circles. Bat skeletal muscle (BM) mitochondria (adult, n=10) are indicated by the dark blue circles. Young mouse brain (YMB) mitochondria aged 4-11 weeks (n=10) and aged mouse brain mitochondria (OMB) aged 78 weeks (n=10) are denoted by orange and red circles respectively. Young mouse skeletal (YMM) muscle mitochondria aged 4-11 weeks (n=9) and aged mouse skeletal muscle mitochondria (OMM) aged 78 weeks (n=10) are indicated by the yellow and blue circles, respectively.

Previously we have reported that mitochondrial lipid profiles are tissue and age specific [22]. We wished to see how the mitochondrial lipid profile from this exceptionally long-lived species compared with the mouse. Orthogonal partial least square-discriminant analysis (OPLS-DA) was performed on the lipidomics data derived from bat and mouse brain and skeletal muscle mitochondria (Figure 2). There are distinct lipid compositional profiles obtained from the mitochondria from the two mammals and also from the two different tissue types. The three groups of muscle data are clustered though there is a distinct separation of the young (YMM) and older mouse muscle (OMM) lipid profiles. It appears that the YMM profile is relatively closer to that of the bat muscle (BM). For the brain profiles on these axes we find that the young and old brain profiles are close in the mouse. The bat brain lipids can be seen to cluster in the same (lowest) section of the plot as the bat muscle samples. Therefore, *species* can now be added to age and tissue type as a determinant of mitochondrial lipid profile [22].

### Ceramide tops the list of the top ten brain mitochondrial lipids with different abundances between bat and mouse

**Figure 3:**
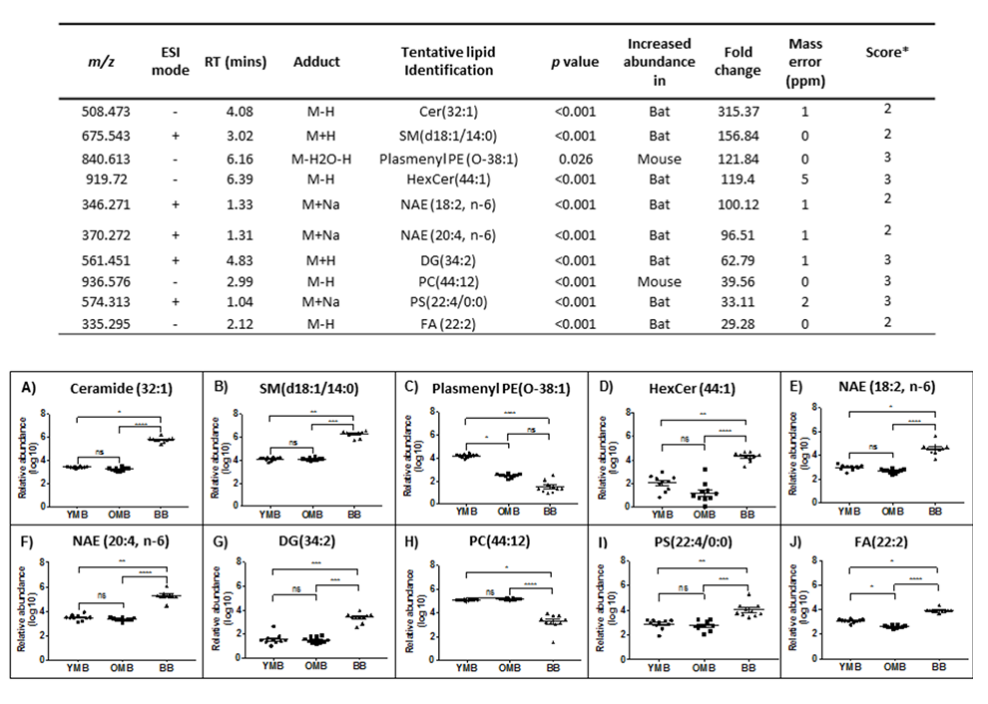
**The ten most different lipid abundance scores in our comparison between bat and mouse brain mitochondrial lipidomes.** m/z – mass charge ratio, ESI mode – electrospray ionisation mode, RT – run time. Confidence scores calculated as per Sumner et al., 2007 [51]. YMB – Young Mouse Brain, OMB – Old Mouse Brain, BB – Bat Brain. Cer – Ceramide, SM – Sphingomyelin, Plasmenyl PE – Plasmenylphosphatidylethanolamine, HexCer – Hexosylceramide, NAE- *n-*acylethanolamine, PS- phosphatidylserine, FA- fatty acid, DG - diacylglyceride and PC- phosphatidylcholine. ns p>0.05, * p<0.05, ** p<0.01, *** p<0.001, **** p<0.0001.

**Figure 4:**
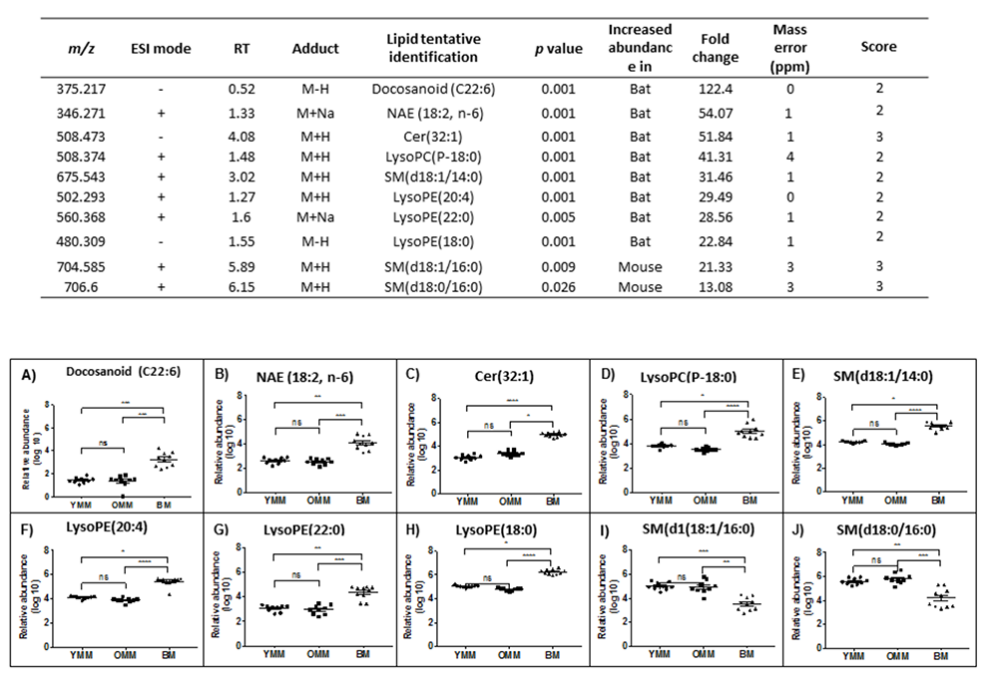
**The ten most different lipid abundance scores in our comparison between bat and mouse skeletal muscle mitochondrial lipidomes.** m/z – mass charge ratio, ESI mode – electrospray ionisation mode, RT – run time. Confidence scores calculated as per Sumner et al., 2007 [51]. YMM – Young Mouse Muscle, OMM – Old Mouse Muscle, BM – Bat Muscle. Cer – Ceramide, NAE- *n-*acylethanolamine, SM – Sphingomyelin, Lyso PE – Lyso-phosphatidylethanolamine and Lyso PC - Lysophosphatidycholine. Ns p>0.05, * p<0.05, ** p<0.01, *** p<0.001, **** p<0.0001.

Having confirmed that there are differences in the lipid composition of the bat compared with mouse mitochondria, we focused on the lipids that had the greatest discrepancies between the two mammals. We identified the top 10 lipids with greatest overall fold change between bat and mouse brain mitochondria (Figure 3). We also identified the top 10 lipids with the greatest fold difference between the bat and mouse skeletal muscle mitochondria (Figure 4). When comparing brain mitochondrial lipids between species, ceramide (32:1) showed the biggest difference (315 fold) and highly significant between mouse and bat brain mitochondria (Figure 3A). There was not a significant difference between the YMB and OMB ceramide (32:1). The lipids showing the biggest discrepancies between bat and mouse brain mitochondria are plotted and show highly significant differences (Figure 3). A similar species identification process provided a top ten list of lipids with changed abundance in skeletal muscle mitochondria. In this case the greatest difference is docosanoid (C22:6) species (122 fold).

### N-acylethanolamine species are more abundant in bat mitochondria

We looked to see whether any particular groups of lipids could be associated with one species or the other. Several N-acylethanolamine species were found in both the top ten muscle and brain lists (Supplementary Table 2 and Figures 3 and 4). In every case, these potentially bioactive fatty acid derivatives, involved as mediators of inflammatory processes are found to be significantly more abundant in the bat mitochondrial preparations. Taking our overall findings together it appears that the most significantly more abundant lipids in bat mitochondria include docosanoids, ceramides, N-acylethanolamines, docosahexaenoic acid (DHA), docosapentaenoic acid (DPA) and arachidonic acid.

### Fatty acids are also comparatively more abundant in bat mitochondria

**Figure 5:**
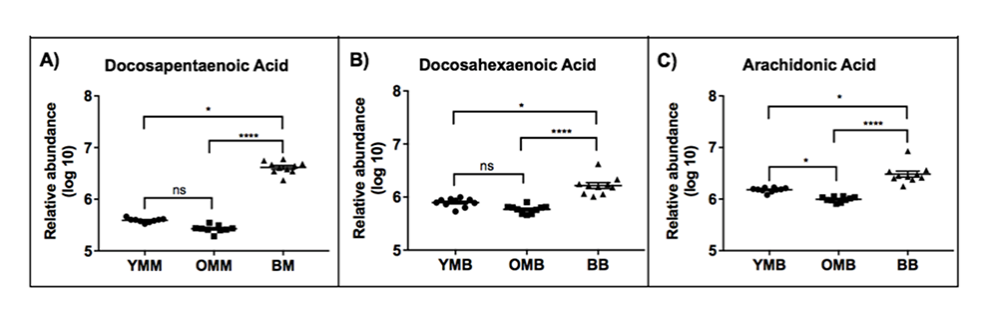
**We observed a preponderance of free fatty acids which were more abundant in the bat mitochondria across both tissue types.** Docosapentaenoic acid, Docosahexaenoic acid and Arachidonic acid were each found to be significantly higher abundance in both bat muscle and brain mitochondria. YMB – Young mouse muscle, OMM – Old mouse muscle, OBM – Old bat muscle, YMB – Young Mouse Brain, OMB – Old Mouse Brain, BB – Bat Brain. ns p>0.05, * p<0.05, ** p<0.01, *** p<0.001, **** p<0.0001. See Supplementary Table 5 for statistical tests and values. Stats: Kruskall-Wallis to compare between Bat, young muscle and old muscle (three groups). Then a Dunn’s multiple comparisons test to compare between two groups.

Another characteristic and important group of mitochondrial lipids that largely characterise bat mitochondria were the fatty acids. We identified 43 different fatty acids that have different abundance in the bat and the mouse mitochondria (Supplementary Tables 3 and 4). Interestingly some of the identified lipids some were found to be odd carbon number fatty acids, for instance C15 and C17. Mammals do not endogenously synthesise odd chain fatty acids and so these are likely to reflect the bat insectivorous diet. Odd carbon number fatty acids are highlighted in Supplementary Tables 3 and 4 since they are considered unusual but may be connected with health measures [25]. We found that all 43 identified fatty acids are more abundant in the bat brain mitochondria (Supplementary Table 3). There were greater levels of all 23 identified PUFAs in the bat brain mitochondria, 13 of these were significantly higher in abundance in the bat brain compared to the mouse brain (*p*<0.05) (Figure 5).

Skeletal muscle lipids follow a similar trend where 36 of the 43 identified fatty acids are more abundant in the bat mitochondria compared to the mouse (Supplementary Table 4). Of the lipids that were higher in the bat skeletal muscle than the mouse muscle mitochondria, 20 were identified as long chain fatty acids C20 and above.

### Mitochondrial lipid binding proteins and fatty acids may modulate lifespan

**Figure 6:**
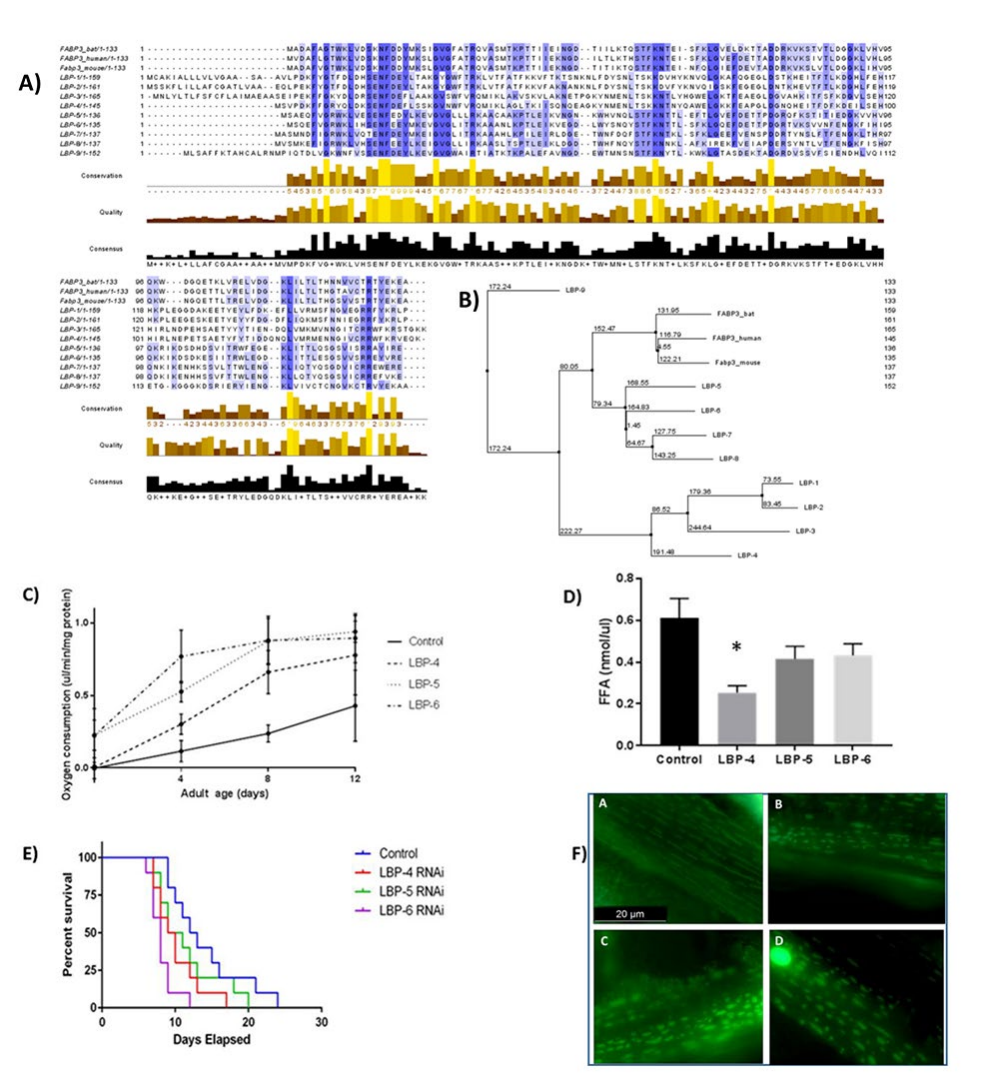
**Fatty acid binding protein orthologues in *C. elegans* confirm the link between these proteins, fatty acid levels, mitochondrial function and lifespan.** (**A**) Sequence alignment of FABP3 and *C. elegans* orthologues LBP-4, -5 and -6. A long chain fatty acid transporter protein in *H. sapiens, M. musculus* and *C. elegans*. Multiple sequence alignment of three *C. elegans* lipid binding protein family members, LBP-4, -5 and -6, with fatty acid binding protein 3 from *H. sapiens*. Amino acid residues are coloured shades of blue signifying their sequence homology; lightest is 30%, darkest is 100%. Sequence homology is 23.74%, 45.52% and 48.15% for LBP-4, -5 and -6 respectively. Sequence alignment was carried out using *Jalview.* (**B**) A phylogenetic tree was produced using PAM 250 scoring to relate human, mouse and *C. elegans* FABP3 orthologues. See Supplementary Table 1 for scoring. (**C**) Oxygen consumption measurements were made on LBP adult knock down adult worms at 4, 8 and 12 days and compared with controls. Reduction of LBP levels caused changes in oxygen consumption in each group compared with controls. The most marked difference in oxygen consumption was recorded in the LBP6 knockdown at days 4 and 8. (**D**) LBP-4, -5 and -6 knockdowns significantly reduce lifespan. *C. elegans* treated with LBP-4, -5 or -6 RNAi show significant reduction in lifespan p=0.0022 (Log rank test). Median lifespans were 9.5, 10 and 8 days for LBP-4, -5 and -6 respectively. 10 worms were followed for each treatment. (**E**) Measurement of free fatty acid quantities in the LBP knock downs confirmed a reduction in free fatty acid abundance in each of the orthologue knock-downs, reaching significance in LBP-4 (p value = 0.027). (**F**) LBP-4, -5 and -6 knockdown affect mitochondrial form and function. Worms carry the transgene ccIs4251 with GFP fusion proteins localised to the body muscle mitochondria and nuclei. Oxygen consumption in worms at different age points was measured using a *Loligo systems^TM^* electrode array (E). CB5600 worm mitochondria subjected to knock-down of LBP-4, -5 and -6. WT worms grown on OP50 (A). Mitochondria become condensed and disordered when subjected to RNAi for LBP-4 (B), -5 (C) and -6 (F).

In order to see whether our protein findings concur with lipidomics data we chose to focus on a potential mitochondrial role for FABP3 (found to be significantly more abundant in bat skeletal muscle mitochondria) in regulating mitochondrial lipids and thereby lifespan. This work was done in the nematode worm to facilitate monitoring of lifespan changes. We identified fatty acid binding protein orthologues in *C. elegans.* Sequence homology and phylogeny confirm their similarity to FABP3 (Figure 6A and B and Supplementary Table 1). RNAi knockdowns of each of LBP4, 5 and 6 were generated and their lifespans recorded. The LBP6 knockdown worms appear to have a shortened lifespan (Figure 6E). Measurement of free fatty acid quantities in the LBP knock downs confirmed a reduction in free fatty acid abundance in each of the orthologue knock-downs, reaching significance in LBP-4 (p value = 0.027).

### Knock-down of lipid binding proteins alters mitochondrial activity and morphology

To investigate whether reduction of lipid binding proteins has a mitochondrial effect, the knockdown worms and controls were placed in an oxygen electrode array (Loligo Systems^TM^) and oxygen consumption rates measured at different ages. The knock-downs all had a higher oxygen consumption rate when compared with controls. Controls exhibited a higher oxygen consumption rate as they grew older and therefore the mutants have a more similar phenotype to the aged control specimens (Figure 6C).

In order to visually assess mitochondrial health and organisation, worms expressing GFP in mitochondria and nuclei (CB500) were used for the LBP4, 5 and 6 knock-down experiments. We visualised the worms alongside controls to compare mitochondrial morphology (Figure 6F). With each of the knock-downs it is apparent that the mitochondria are fragmented and there is an organisational change from the long mitochondrial networks found in the control.

## DISCUSSION

We set out to examine mitochondrial compositional differences between the mouse and relatively long-lived pipistrelle bat. To focus our study, we examined enriched mitochondrial fractions extracted from whole brain and skeletal muscle, these were chosen as tissue types that decline in performance with age and are likely to exhibit lifespan regulated changes. The different metabolic nature of these tissues can give us a broader view of age-related mitochondrial changes.

Identification of proteins that are present in different relative quantities when comparing mouse and bat suggested mitochondrial electron transport chain components are interesting in this context. The electron transport chain proteins are the machinery for generating ATP and changes in these molecules could be postulated to alter the efficiency of intracellular energy production. In brain mitochondria we also found significant differences in two RAS oncogene family RAB proteins 1B and 14, these are GTPases involved in intracellular trafficking between Golgi and endosomes [26,27], there is accumulating evidence that differential regulation of tumour pathway proteins occurs also in species of bats [28–30]. In the skeletal muscle we found many mitochondria specific protein differences. However, two of the proteins significantly altered between the mouse and bat skeletal muscle are best known for their distribution in cardiac muscle, namely myosin light chain 2 and fatty acid binding protein 3 (FABP3) [31,32]. Myosin light chain 2 has been found to have half the levels of phosphorylation in the naked mole rat when compared with the mouse, which makes this an especially interesting protein in exceptionally long-lived species [33]. We have now confirmed that FABP3 is much more abundant in the bat muscle mitochondria. FABP3 and other members of the FABP family have been shown to transport fatty acids and other lipids intracellularly [32]. Our finding that FABP3 is upregulated in bat mitochondria is reflected in our lipidomics dataset from the same tissue types. This makes it reasonable to postulate that increased quantities of fatty acids and their transport are a feature of the exceptional physiology of the bat.

To examine mitochondrial lipid differences between bat and mouse we used ultra-high performance liquid chromatography coupled with high-resolution mass spectrometry to separate and identify lipid contents of mitochondrial fractions from brain and muscle. Ceramide (32:1), N-acylethanolamines (18:2 and 22:6) and a docosanoid species were identified within the top six lipid species, each more abundant in the bat mitochondrial fractions. We noticed that lipid species known to be metabolic precursors of lipid mediators of inflammatory processes, particularly the N-acylethanolamines, DPA, DHA and AA are found to be highly abundant in bat mitochondria when compared with young or older mouse mitochondrial lipids [34,35]. This finding may support the idea of a central function for these lipids in the longer maintenance of healthy mitochondria. Importantly these identified lipids can each be transported by FABP3 which was shown to be upregulated in the bat muscle fractions [36]. Our data measuring higher levels of DHA in the bat mitochondria appear to contrast with data from membranes isolated from naked mole-rat which have been found to contain lower levels of DHA [37]. It was suggested the lower levels of DHA are protective in the mole-rat since this reduces the potential for lipid peroxidation. Therefore, our observations either reveal an alternative protective strategy where larger quantities of DHA offset the functional losses through peroxidation, or alternatively that whole mitochondrial content cannot necessarily be compared with isolated membrane fractions. It should also be considered that the two different mammals have very different diets. Naked mole-rats eat roots and tubers whereas the bats we are studying are insectivorous. There is a large body of data showing the beneficial effect of dietary DHA on ageing tissues and membranes therefore large quantities could be perceived as protective [34,38].

In order to interrogate the effect of free fatty acids upon mitochondrial health we interrogated FABP orthologues (Lipid Binding Proteins, LBPs) in the nematode worm *C. elegans,* an animal model that has been used extensively to study pathways important in ageing [39,40]. We were able to confirm that knock down of LBPs cause a significant reduction in free fatty acid content (LBP4) and lifespan (LBP6). Previously another group has shown that the presence of lipid binding protein LBP-8 is an important chaperone regulating longevity in *C. elegans* [41]. Our knock-down of LBPs also affected mitochondrial function neatly supporting a previous work where mutation of LBP-5 was found to increase glycolysis, ostensibly to mitigate mitochondrial dysfunction [42]. We suggest that FABP3 is a modulator of mitochondrial health and we find higher levels of this protein are associated with increased levels of beneficial lipid species. Much of the research connected with FABP3 (often referred to as heart type fatty acid binding protein) is connected with cardiac function. In zebrafish a FABP3-morpholino (MO)-induced apoptosis and mitochondrial dysfunction in cardiac development [43]. In humans, FABP3 gene expression in the atrium was reduced in patients with post-operative atrial fibrillation. These findings suggest a potential link between altered fatty acid transport in the atrium and increased atrial fibrillation onset after cardiac surgery [44]. In muscle, reduced FABP3 is associated with Myostatin gene deletion in mice and causes metabolic changes with decreased mitochondria content, disturbance in mitochondrial respiratory function and increased muscle fatigability [45].

It has been shown that polyunsaturated fatty acids (PUFAs) are essential for brain development and function. Increasing evidence shows that an imbalance of PUFAs is associated with psychiatric disorders, including autism and schizophrenia. Fabp3 KO mice have cognitive and emotional deficits and FABP3 regulates GABA synthesis through transcriptional regulation of *Gad67* [46]. Interestingly, *Gad67* is upregulated in the retina of the Klotho knockout mouse model of accelerated ageing [47]. The importance of *Fabp3* in non-cardiac tissues is further supported by work in the klotho knockout mouse, where significantly decreased levels of *Fabp3* are measured in the submandibular salivary gland [48].

We show that decreased quantities of FABP3 orthologues are detrimental to mitochondrial health. The literature supports this as a mechanism associated with mitochondrial dysfunction across many tissues. Mechanisms to increase levels of FABP3 or fatty acids in the mitochondrial compartment may be of interest in supporting mitochondrial health through the lifespan.

## MATERIALS AND METHODS

### Ethics and tissues

This study has been approved by the School of Veterinary Medicine and Science Ethics and Clinical Review Panel, University of Nottingham (ERN# 1922 170103). All mouse tissues were obtained from Charles River Laboratories. Mice were humanely killed by a trained individual using an approved schedule 1 method, in full accordance with UK Home Office guidelines. Human brain tissues were obtained (after ethical review) from the Parkinson’s UK Brain Bank in London.

### Tissue and mitochondrial preparation

Bat tissues were obtained from animals that had died at the hospital from natural causes and adult individuals were selected for likely older age by an experienced hospital bat handler based on previous observations that older adults have a rougher coat condition with thinning patches and other general signs such as tattered wing and ear skin. Bat carcasses were obtained from the West Yorkshire Bat Hospital with permission from *Natural England*. It was important to ensure that tissues from the bats were obtained in good condition for our biochemical analyses. Bat carcasses were frozen as soon as possible after death and transported on dry ice to the laboratory where mitochondrial extractions were performed. Tissue integrity for the bat tissues was confirmed for another study where high >8 RIN (RNA integrity) values were obtained for RNA extracted from these bat samples. Figure 1F shows also that mitochondrial Complex I activity is not recorded as significantly different from the mitochondria harvested from mouse tissues. We can therefore be confident that both molecular and functional integrity were preserved in the harvested bat tissue specimens. All mouse tissues were obtained from *Charles River Laboratories.* Mice were humanely killed by a trained individual using an approved schedule 1 method, in full accordance with *UK Home Office* guidelines. The mitochondrial fraction was isolated by differential centrifugation from the tissues as previously described [49].

### Mitochondrial preparations

Brain and skeletal muscle tissue were dissected from young C57BL/6J mice (4-11 weeks old), old C57BL/6J mice (78 weeks old) (Charles River) and from adult *Pipistrellus pipistrellus* bats (>12 months old). (Further information in Supplementary Methods).

### Mitochondrial lipid extraction

Mitochondrial fractions were prepared from mouse brain tissue (young 4-11 weeks old n=10 and old 78 weeks n=10), mouse skeletal muscle tissue (young 4-11 weeks n=10 and old 78 weeks n=10), adult bat brain (n=10) and adult bat skeletal muscle tissue (n=10). 100 μl of chloroform/methanol (2:1) was added to each sample and extracted for 20 min using a multiplace vortex before undergoing centrifugation (1300 rpm, 10 min) at 4°C. The supernatants were removed to HPLC vials. Isopropanol (50 μl) was added to each vial before lipidomics analysis.

### Lipidomics analysis using liquid chromatography coupled to high resolution mass spectrometry

Ultra-high performance liquid chromatography coupled with high resolution mass spectrometry (UHPLC-HRMS) was performed on the mitochondrial lipid extracts (10 µL injection volume) using an Accela LC coupled with an Exactive mass spectrometer (ThermoFisher Scientific, Waltham, USA) in positive and negative electrospray ionisation modes (ESI).

### Fluorescence imaging - GFP-tagged muscle mitochondria and nuclei

CB5600 (*ccls4251 (Pmyo-3::Ngfp-lacZ; Pmyo-1::Mtgfp)* I; *him-8 (e1489)* IV) with GFP fusion proteins localised to mitochondria and nuclei [50]. Worms were age-synchronised using standard methods and grown for 48-60 hours at 20°C until they reach L4 stage. 20 worms from the plates were picked into 20µl M9 on a microscope slide. Coverslip was applied and worms were imaged using the Leica CTR5000 under a GFP filter (460-500nm, blue light excitation) at 100X magnification.

### O2 consumption trials

A 24-channel optical fluorescence, oxygen sensing system (Loligo Systems, Copenhagen, Denmark) was used for the analysis of oxygen consumption upon knockdown of LBP-4, -5 and -6 across the lifespan of adult *C.elegans*. (For detail please see Supplemental Methods)

### Free fatty acid (FFA) analysis

Total fatty acid concentration was measured using the Free Fatty Acid Quantification Kit (AB65341, Abcam), according to manufacturer’s instructions. Briefly, 4 plates of cleaned adult age day 0 worms were suspended in chloroform/Triton X-100 (1% Triton X-100) and homogenised by 30 strokes of a 1.5ml mini-pestle, vortexed and centrifuged at 16,000g for 10 min. Lower phases of the samples were collected and vacuum dried for 30 min. Reported concentrations were quantified by fluorometric analysis (Varioskan LUX Multimode Microplate Reader).

### Data analysis see Supplementary Methods.

## SUPPLEMENTARY MATERIAL

Supplementary File
