# Supplementary material for: A comparison of the mitochondrial proteome and lipidome in the mouse and long-lived Pipistrelle bats
Source: Aging (Albany NY). 2019 Mar 19;11(6):1664–85. doi: 10.18632/aging.101861 (PMC6461166; doi:10.18632/aging.101861)
Supplement: Supplementary File [file aging-11-101861-s001.pdf]

SUPPLEMENTARY MATERIAL

Supplementary Methods

Mitochondrial sub-fractionation

Enriched mitochondrial fractions from human and mouse brain tissues were isolated by differential centrifugation and subjected to a series of ultra-centrifugation steps. Human brain tissues were obtained (after ethical review) from the Parkinson's UK Brain Bank in London. Sub-fractions were analysed by western blotting and purity of the mitochondrial components separated was confirmed using antibodies specific to each sub-fraction – as described previously [1].

2D gel analysis

Mitochondrial samples, mouse brain (n=8), bat brain (n=4) mouse skeletal muscle (n=6), bat skeletal muscle (n=6), underwent iso-electric focussing using ZOOM IPG (Life Technologies) system and pH 3-10 (non-linear) ZOOM IPG strips as described by Pollard *et al* 2016 [2].

Due to the large differences between the mouse and bat skeletal muscle mitochondrial proteome, an additional 2D gel was run containing both mouse and bat skeletal muscle mitochondria to provide a reference image in SameSpots software. Protein spots with a *p value* of less than 0.1 and a fold change greater than 1.2 were further analysed (one-way ANOVA). Proteins identifications were obtained as previously described [1].

Western blot

Western blotting was carried out as described previously [1]. Antibody dilutions: Fatty acid binding protein 3, PA5-13461 (ThermoFisher Scientific) 1:1250 dilution in 5% (w/v) BSA in 1xTBS-T; GAPDH G9545 (SIGMA) 1:5000 dilution in 3% (w/v) BSA in 1xTBS-T. Muscle mitochondrial samples were normalised to GAPDH level. The average of three samples for each condition (bat and mouse) were plotted showing the mean +/- SEM. Statistical analyses were carried out in GraphPad Prism.

Lipidomics analysis using liquid chromatography coupled to high resolution mass spectrometry – detail

An ACE EXL Excel 2 µm Super C18 2.1 x 50 mm column equipped with an appropriate guard column was maintained at 40°C with a stepped flow rate. The LC gradient program used a water (60%) acetonitrile (40%) (A)-to-water (10%)/acetonitrile (100%)/isopropanol (80%) gradient (B) modified with 0.1% ammonium acetate (gradient 30%-100 %B over 12 min). Ions were monitored within the range of *m/z* 100 to 1500 (ESI voltage: 3500, capillary temperature: 350°C, scan rate: 250 ms, FT resolution: 25,000). A pooled quality control sample comprising 5 µL from each experimental sample was generated and injected at regular intervals throughout the run to assess the quality of the analysis. LC-MS data were normalised to total ion count for each sample, aligned and exported using the propriety software Progenesis QI (Nonlinear Dynamics, UK).

Data analysis

All lipids with samples recorded with zero abundance were removed from the dataset. The fold change in abundance between the adult bat and mouse (average of all samples 4-11 weeks and 78 weeks) was calculated for each lipid. The lipids were sorted by fold change. The top 10 lipids with the highest fold change in abundance between the bat and mouse brain mitochondria were selected. The top 10 lipids with the highest fold change in abundance between the bat and mouse skeletal muscle mitochondria were selected. The Kruskall-Wallis test was used to compare between bat, young muscle and old muscle (non-parametric test between three groups). Followed by Dunn's multiple comparisons test to compare between two groups (YMB vs OMB, YMB vs BB, OMB vs BB). GraphPad Prism. Not significant $p>0.05$, * $p<0.05$, ** $p<0.01$, *** $p<0.001$, **** $p<0.0001$. Multivariate analysis-orthogonal partial least square-discriminant analysis (OPLS-DA) was carried out (SIMCA-P 13.0.2 version, Umetrics AB, Umea, Sweden). Lipids were identified by references to accurate masses contained in the Lipid Maps (lipidmaps.org) and Human Metabolome (hmdb.ca) databases. Confidence scores were assigned to the tentative identifications for each lipid in the top 10 tables. Confidences scores were selected based on metabolite identification requirements as described by Sumner et al 2007 [3]

Sequence homology and phylogenetic tree

Multiple sequence alignment containing the sequences of bat, human and mouse FABP3 and *C.elegans* FABP3 orthologues LBP-1 to -9 was created from UCSC genome browser database, accession numbers are given in Supplementary Table 1. Amino acid residues are coloured shades of blue signifying their sequence homology; lightest is 30%, darkest is 100%. Sequence homology data are shown in Supplementary Table 1. Sequence alignment was carried out using Jalview and phylogeny produced using PAM 250 scoring.

O2 consumption trials –detail

The microplate sensor dish reader (SDR) contains individual sensors in each well. Oxygen consumption trials were carried out using 5 worms per well with four replicate wells for each mutant and control. Worms were age-synched as described previously. The first oxygen consumption measurements were conducted on newly adult worms, 48-72 hours after age-synch; termed adult age day 0. Oxygen consumption was measured at 15 second intervals continuously for 3 hours. Additional trials were conducted at adult age days 4, 8 and 12. Briefly, 100ul M9 buffer was added to individual wells and sensor spots at the base of the wells allowed to equilibrate to the buffer for 30 minutes, at 24 degrees Celsius. Worms were moved directly into the buffer. As soon as this was done, a parafilm covered silicon block was placed atop the SDR, creating an oxygen impermeable seal. Control wells of M9 buffer only were used to correct for background levels of respiration. Oxygen consumption over time was calculated using the first 15 minutes of each trial. All data was corrected for volume of buffer and total protein per well, expressed as ul/min/mg protein.

Supplementary Tables

Supplementary Table 1.

Species	Gene	Sequence homology	Accession number
H. sapiens	FABP3		NM_004102
M. musculus	FABP3	86%	uc008uzd.1
Microbat	FABP3	87%	NM_001099931
C. elegans	LBP-1	27.46%	NM_076156.5
	LBP-2	28.85%	NM_076157.7
	LBP-3	21.325	NM_001047784.4
	LBP-4	23.74%	NM_072615
	LBP-5	45.52%	NM_059527
	LBP-6	48.15%	NM_059525
	LBP-7	44.03%	NM_074039
	LBP-8	38.17%	NM_074043
	LBP-9	41.22%	NM_001038422

Supplementary Table 2. Statistical analysis for the N-acyl ethanolamines (NAEs) identified in the top lipid differences in abundance between the mouse and bat mitochondria.

Lipid	Comparison between	Statistical test	*p* value
NAE 18:2	BB/YMB/OMB	Kruskal-Wallis and Bonferroni correction	<0.0001*
	YMB/OMB	Mann-Whitney	0.0039*
	BB/YMB	Mann-Whitney	<0.0001*
	BB/OMB	Mann-Whitney	<0.0001*
NAE 20:4	BB/YMB/OMB	Kruskal-Wallis and Bonferroni correction	<0.0001*
	YMB/OMB	Mann-Whitney	0.0892
	BB/YMB	Mann-Whitney	<0.0001*
	BB/OMB	Mann-Whitney	<0.0001*
NAE 18:2	BM/YMM/OMM	Kruskal-Wallis and Bonferroni correction	<0.0001*
	YMM/OMM	Mann-Whitney	0.2973
	BM/YMM	Mann-Whitney	<0.0001*
	BM/OMM	Mann-Whitney	<0.0001*

Number of samples; bat brain (BB) mitochondrial (adult, n=10), bat skeletal muscle (BM) mitochondria (adult, n=10), young mouse brain (YMB) mitochondria aged 4-11 weeks (n=10), old mouse brain mitochondria (OMB) aged 78 weeks (n=10), young mouse skeletal (YMM) muscle mitochondria aged 4-11 weeks (n=9) and aged mouse skeletal muscle mitochondria (OMM) aged 78 weeks (n=10). Statistical analysis was performed in GraphPad Prism.

Supplementary Table 3. The fatty acids identified were all more abundant in the bat brain mitochondria.

m/z	RT (mins)	Lipid tentative identification	Fatty acid group	Fold change	Score
335.295	2.12	C22:2	PUFA	29.28	2
329.248	1.37	C22:5 Docosapentaenoic acid	PUFA	15.10	2
267.233	1.45	C17:1 Heptadecenoic acid	MUFA	13.60	2
279.233	1.36	Linoelic	PUFA	10.49	2
253.217	1.28	C16:1 Palmitoleic acid	MUFA	10.32	2
305.248	1.47	C20:3 Eicosatrienoic acid	PUFA	9.83	2
333.280	1.85	C22:3	PUFA	9.52	2
365.342	3.22	C24:1	MUFA	9.07	2
297.279	2.22	C19:0	SFA	8.68	2
309.280	2.01	C20:2	PUFA	8.41	2
357.280	1.69	C24:5	PUFA	8.35	2
331.264	1.57	C22:4	PUFA	5.65	2
269.249	1.73	C17:0 Heptadecanoic acid	SFA	5.27	2
281.248	1.61	C18:1 Oleic	MUFA	4.87	2
303.233	1.32	C20:4 Arachadonic acid	PUFA	2.65	2
327.233	1.21	C22:6 Docosahexaenoic acid	PUFA	2.57	2
255.233	1.55	C16:0 Palmitic	SFA	2.75	2
277.217	1.17	C18:3 Linolenic	PUFA	11.13	2
225.186	0.96	C14:1	MUFA	4.78	2
317.248	1.45	C21:5	PUFA	1192.67	2
241.217	1.37	C15:0 Pentadecanoic acid	SFA	3.96	2
339.326	3.20	C22:0	SFA	2.28	2
295.227	0.61	C18 H31 O3	HFA	4.09	2
293.248	1.51	C19:2	PUFA	271.46	2
293.212	0.71	C18 H29 O3	HFA	567.49	2
367.358	4.03	C24:0	SFA	3.22	2
311.295	2.50	C20:1	MUFA	2.13	2
361.311	2.27	C24:3	PUFA	16.25	2
363.327	2.65	C24:2	PUFA	18.28	2
323.295	2.27	C21:1	MUFA	15.43	2
393.373	4.01	C26:1	MUFA	12.27	2
359.295	1.92	C24:4	PUFA	5.87	2
239.201	1.15	C15:1	MUFA	8.64	2
385.311	2.05	C26:5	PUFA	186.51	2
355.264	1.47	C24:6	PUFA	3.22	2
387.327	2.35	C26:4	PUFA	10.27	2
395.389	4.93	C26:0	SFA	2.92	2
325.311	2.84	C21:0	SFA	2.98	2
199.170	0.88	C12 SFFA	SFA	1.57	2
337.236	1.53	C22:1	MUFA	1.69	2

227.201	1.21	C14:0	SFA	1.40	2
391.358	3.34	C26:2	PUFA	30.76	2
313.078	3.28	C20:0	SFA	3.06	2

Odd carbon number fatty acids are considered unusual but may be connected with health measures [4]. Lipids were identified using the human metabolome and Lipid maps databases. Abbreviations: Retention time (RT) in minutes, electrospray ionisation mode (ESI), polyunsaturated fatty acid (PUFA), saturated fatty acid (SFA), monounsaturated fatty acid (MUFA) and hydroxy fatty acid (HFA). All ions are in negative mode. Scores according to Sumner et al., 2007 [3].

Supplementary Table 4. The fatty acids identified in the bat and mouse skeletal muscle mitochondria.

m/z	RT	Tentative lipid Identity	Fatty acid group	Increased expression in	Fold change	Score
293.212	0.71	C18 H29 O3	HFA	Bat	53.32	2
297.279	2.22	C19:0	SFA	Bat	23.78	2
295.227	0.61	C18 H31 O3	HFA	Bat	13.11	2
329.248	1.37	C22:5 Docosapentaenoic acid	PUFA	Bat	10.89	2
269.249	1.73	C17:0 Heptadecanoic acid	SFA	Bat	9.81	2
325.311	2.84	C21:0	SFA	Bat	6.03	2
337.311	2.54	C22:1	MUFA	Bat	5.74	2
327.233	1.21	C22:6 Docosahexaenoic acid	PUFA	Bat	4.70	2
303.233	1.32	C20:4 Arachadonic acid	PUFA	Bat	4.23	2
241.217	1.37	C15:0 Pentadecanoic acid	SFA	Bat	3.41	2
279.233	1.36	Linoelic	PUFA	Bat	3.02	2
309.280	2.01	C20:2	PUFA	Bat	2.89	2
255.233	1.55	C16:0 Palmitic	SFA	Bat	2.72	2
339.326	3.20	C22:0	SFA	Bat	2.49	2
281.248	1.61	C18:1 Oleic	MUFA	Bat	3.59	2
335.295	2.12	C22:2	PUFA	Bat	4.05	2
367.358	4.03	C24:0	SFA	Bat	1.96	2
393.373	4.01	C26:1	MUFA	Bat	32.18	2
365.342	3.22	C24:1	MUFA	Bat	2.01	2
267.233	1.45	C17:1 Heptadecenoic acid	MUFA	Bat	3.90	
385.311	2.05	C26:5	PUFA	Mouse	5.78	2
317.248	1.45	C21:5	PUFA	Bat	166.35	2
227.201	1.21	C14:0	SFA	Bat	1.46	2
323.295	2.27	C21:1	MUFA	Bat	2.90	2
359.295	1.92	C24:4	PUFA	Mouse	1.63	2
331.264	1.57	C22:4	PUFA	Bat	3.04	2
363.327	2.65	C24:2	PUFA	Bat	1.88	2
333.280	1.85	C22:3	PUFA	Mouse	1.23	2
293.248	1.51	C19:2	PUFA	Bat	6.57	2
311.295	1.77	C20:1	MUFA	Mouse	1.64	2
225.186	0.96	C14:1	MUFA	Bat	10.34	2
199.170	0.88	C12 SFFA	SFA	Bat	2.29	2
277.217	1.17	C18:3 Linolenic	PUFA	Bat	2.97	2
387.327	2.35	C26:4	PUFA	Mouse	1.05	2
239.201	1.15	C15:1	MUFA	Bat	3.68	2
395.389	4.93	C26:0	SFA	Mouse	1.00	2
313.078	3.28	C20:0	SFA	Mouse	100.36	2
253.217	1.28	C16:1 Palmitoleic acid	MUFA	Bat	1.87	2
391.358	3.34	C26:2	PUFA	Bat	1.89	2
361.311	2.27	C24:3	PUFA	Bat	1.22	2
355.264	1.47	C24:6	PUFA	Bat	1.07	2
305.248	1.47	C20:3 Eicosatrienoic acid	PUFA	Bat	1.10	2

357.280	1.69	C24:5	PUFA	Bat	1.64	2

Odd carbon number fatty acids are considered unusual but may be connected with health measures [4]. Lipids were identified using the human metabolome and Lipid maps databases. Abbreviations: Retention time (RT) in minutes, electrospray ionisation mode (ESI), polyunsaturated fatty acid (PUFA), saturated fatty acid (SFA), monounsaturated fatty acid (MUFA) and hydroxy fatty acid (HFA). All ions are in negative mode. Scores according to Sumner et al., 2007 [2].

Supplementary Table 5. Statistical analysis for the representative fatty acids presented in Figure 5 (D,E,F).

Lipid	Comparison between	Statistical test	*p* value
Docosahexaenoic acid	BB/YMB/OMB	Kruskal-Wallis and Bonferroni correction	<0.0001*
	YMB/OMB	Mann-Whitney	0.0052*
	BB/YMB	Mann-Whitney	<0.0001*
	BB/OMB	Mann-Whitney	<0.0001*
Arachidonic acid	BB/YMB/OMB	Kruskal-Wallis and Bonferroni correction	<0.0001*
	YMB/OMB	Mann-Whitney	<0.0001*
	BB/YMB	Mann-Whitney	<0.0001*
	BB/OMB	Mann-Whitney	<0.0001*
Docosapentaenoic acid	BM/YMM/OMM	Kruskal-Wallis and Bonferroni correction	<0.0001*
	YMM/OMM	Mann-Whitney	<0.0001*
	BM/YM	Mann-Whitney	<0.0001*
	BM/OMM	Mann-Whitney	<0.0001*

Number of samples; bat brain (BB) mitochondrial (adult, n=10), bat skeletal muscle (BM) mitochondria (adult, n=10), young mouse brain (YMB) mitochondria aged 4-11 weeks (n=10), old mouse brain mitochondria (OMB) aged 78 weeks (n=10), young mouse skeletal (YMM) muscle mitochondria aged 4-11 weeks (n=9) and aged mouse skeletal muscle mitochondria (OMM) aged 78 weeks (n=10). Statistical analysis was performed in GraphPad Prism.

Supplementary References

1. Shephard F, Greville-Heygate O, Marsh O, Anderson S, Chakrabarti L. A mitochondrial location for haemoglobins-Dynamic distribution in ageing and Parkinson's disease. Mitochondrion. 2013; 14: 64–72.

2. Pollard A, Shephard F, Freed J, Liddell S, Chakrabarti L. Mitochondrial proteomic profiling reveals increased carbonic anhydrase II in aging and neurodegeneration. Aging (Albany NY). 2016; .

3. Sumner LW, Amberg A, Barrett D, Beale MH, Beger R, Daykin CA, Fan TW-M, Fiehn O, Goodacre R, Griffin JL, Hankemeier T, Hardy N, Harnly J, et al. Proposed minimum reporting standards for chemical analysis Chemical Analysis Working Group (CAWG) Metabolomics Standards Initiative (MSI). Metabolomics. NIH Public Access; 2007; 3: 211.

4. Jenkins B, West JA, Koulman A. A review of odd-chain fatty acid metabolism and the role of pentadecanoic Acid (c15:0) and heptadecanoic Acid (c17:0) in health and disease. Molecules. 2015; 20: 2425–44.
